# Perspectives on Genetic Research: Results From a Survey of Navajo Community Members

**DOI:** 10.3389/fgene.2021.734529

**Published:** 2021-12-02

**Authors:** Katrina G. Claw, Nicolas Dundas, Michael S. Parrish, Rene L. Begay, Travis L. Teller, Nanibaa’ A. Garrison, Franklin Sage

**Affiliations:** ^1^ Division of Biomedical Informatics and Personalized Medicine, Colorado Center for Personalized Medicine, University of Colorado Anschutz Medical Campus, Aurora, CO, United States; ^2^ Treuman Katz Center for Pediatric Bioethics, Seattle Children’s Hospital and Research Institute, Seattle, WA, United States; ^3^ Diné Policy Institute, Tsaile, AZ, United States; ^4^ Centers for American Indian and Alaska Native Health, University of Colorado Anschutz Medical Campus, Aurora, CO, United States; ^5^ Institute for Society and Genetics, College of Letters and Science, University of California, Los Angeles, CA, United States; ^6^ Institute for Precision Health, David Geffen School of Medicine, University of California, Los Angeles, CA, United States; ^7^ Division of General Internal Medicine and Health Services Research, David Geffen School of Medicine, University of California, Los Angeles, CA, United States

**Keywords:** Navajo, genetics, ethics, health policy, Diné, Navajo Nation, American Indian, Indigenous

## Abstract

The Navajo Nation placed a moratorium on genetic research studies in 2002, in part due to concerns about historical distrust, exploitation, limited expertise and resources, and the lack of a genetics policy. Navajo tribal leaders, scientists, and policy experts are exploring the possibility of lifting the moratorium, developing a genetic research policy, and discussing its potential health implications. This study aimed to identify the key concerns, needs, and desires of Navajo people regarding genetic research. We conducted a survey of Navajo individuals to assess knowledge of the moratorium and research, gauge interest in genetic research, and quantify appropriate genetic research topics to understand broad views and concerns. We performed descriptive statistics and tested associations between relevant categorical variables using Chi-square tests. We hypothesized that individuals with more knowledge about the moratorium and health research increased the likelihood of supporting and participating in genetic research. A total of 690 surveys from Navajo respondents were analyzed. Of these, 63% of respondents reported being unaware of the Navajo Nation’s moratorium on genetic research. There were positive associations between those who knew about the moratorium and willingness to donate biospecimens for research under certain conditions, such as community involvement, review and approval by community leaders, research on diseases affecting the community, and support for lifting the moratorium (*p*-values < 0.001). We found no significant differences between age, gender, religious/spiritual beliefs, or agency affiliation with knowledge levels of genetics and related topics, participation in relation to beliefs, and donation of biospecimens. Interestingly, respondents who resided off the Navajo Nation were positively associated with having knowledge of the moratorium, having heard of discussions of genetics on the Navajo Nation, and the lawsuit filed by the Havasupai Tribe. Most respondents agreed that it was very important to develop a policy that incorporates cultural knowledge (56%), is beneficial (56%), and has data sharing protections (59%) before allowing genetic research on the Navajo Nation. Overall, a large proportion of respondents (46%) were unsure about lifting the moratorium and instead wanted more genetics education to assess its potential implications. The study results can inform the direction of future guidelines and policies.

## Introduction

Genetic and genomic science continues to expand rapidly, providing opportunities to address human disease and reduce health disparities. Despite its rapid growth, Indigenous people worldwide are starkly underrepresented in genetic research studies, comprising less than 0.05% of the representation (Bustamante et al., 2011; Popejoy and Fullerton, 2016; Mills and Rahal, 2019), and there has been limited research on disease, treatment, and prevention related to genetic research (Need and Goldstein, 2009). Reasons for these low participation rates are many and relate to ethical and sociopolitical factors (Jacobs et al., 2010; Claw et al., 2018; Garrison et al., 2019; Caron et al., 2020). As genetic and genomic research continues to flourish due to technological advancements, little is known about the perceptions of and knowledge about genetic research in tribal communities, in part due to the lack of equitable inclusion of Indigenous people in the United States (US).

Within the US, Indigenous people (American Indian and Alaska Native people) experience more health disparities compared to the general population (Indian Health Service, 2019). For Navajo people (known as the Diné, or “The People”), an Indigenous tribe located in the southwestern part of the US, some of these health disparities may be attributed to genetic diseases and conditions (Lynch et al., 1994; Erickson, 1999; Li et al., 2002; Begay et al., 2020). More specifically, Navajo people have been subject to a number of genetic research studies on bacterial or viral genetics, blood and human leukocyte antigens, complex diseases, forensics, hereditary diseases, and population genetics and human migration since 1926 (Begay et al., 2020). The Navajo Nation (NN) is the largest tribe in the US with 399,494 enrolled citizens (Romero, 2021) and the land base spans over 27,000 square miles across Arizona, Utah, and New Mexico. The NN is divided into five agencies and further divided into 110 Chapter districts with 1-4 communities per district, with 5,000 to 8,000 people living in the largest towns. According to an analysis of the 2010 Census data by the Navajo Epidemiology Center, approximately 150,000 people live on the NN (Navajo Division of Health Navajo Epidemiology Center, 2013). Many Navajo people live in adjacent communities that border the NN, commonly referred to as “border towns” (33,000) or in “metro” (86,000) areas (Navajo Division of Health Navajo Epidemiology Center, 2013), thus many members are transitory or commute to and from the NN daily or weekly for economic and social reasons.

The Navajo Nation Human Research Review Board (NNHRRB), which was established in 1996 to review research studies involving Navajo people, raised questions and concerns related to inquiries about and the governance of potential genetic research protocols. In April 2002, the Navajo Nation’s Health and Human Services Committee approved a “moratorium on genetic research studies conducted within the jurisdiction of the Navajo Nation until such time that a Navajo Nation Human Research Code has been amended by the Navajo Nation Council” after consulting with tribal leaders, traditional healers, and Navajo people with medical and scientific training (Gift of Life, 2002). A driving factor was a concern about the lack of existing policies or guidelines for genetic research protocols to prevent research harms while respecting the values of Diné culture (Gift of Life, 2002).

After the establishment of the moratorium, very few public discussions have taken place among Diné people about emerging genetic technologies and its associated ethical dimensions. In 2017, the Tribal Data Sharing and Genetics Policy Development Workshop was held at the University of New Mexico (NIH Tribal Health Research Office, 2017) to convene Navajo government leaders, US government agencies, researchers, and stakeholders to discuss genetics, data sharing, future use and storage of biospecimens, integration of traditional knowledge, and tribal sovereignty in the context of a potential policy on the NN. These meetings culminated in the formation of a working group approved by the Navajo Nation’s Health, Education and Human Services Committee and the Naabikíyáti’ Committee (both standing committees of the Navajo Nation Office of Legislative Services), known as the Navajo Nation Genetics Policy Development Working Group, consisting of leaders, scientists, and policy experts from the NN to re-examine the moratorium and develop a policy on genetic research that recognizes the Navajo Nation’s sovereignty, is inclusive of Navajo culture, and builds capacity for oversight of genetic research (Navajo Nation Council, 2018).

Despite ongoing conversations, no prior empirical research has been conducted that identifies the key concerns and perspectives among Navajo citizens regarding genetic research, so the data needed to formulate a well-informed policy were lacking. To this end, a survey was conducted to assess public perceptions of Navajo people about genetic research, identify areas of concern, and solicit feedback on types of genetic research to prioritize or avoid as part of a larger study of perspectives about genetics. Findings from this survey will build a foundation for Navajo tribal policy makers and the general public to make informed decisions regarding appropriate protections against harms and misuse to enable the tribe to benefit from potential genetic research appropriate and respectful research protocols.

## Materials and Methods

### Study Population

A convenience sampling method was used to recruit participants who identify as Navajo, mainly by in-person attendance of study team members at public meetings and events held on or near the NN (e.g., four NN Agency Council meetings and other public forums) and house-to-house visits in three communities in Northern Agency and three satellite communities in New Mexico. Additional participants were recruited via social media, local newspapers, and email invitation to complete an online version of the survey. A raffle drawing with 20 gift card prizes worth $25.00 were offered to incentivize people to participate in the survey each. Contact information was collected separately and discarded after the raffle drawing. Participants indicated consent by completing the survey, and no identifying data from respondents were linked to the surveys.

### Survey Development and Administration

The survey results reported here are a part of a larger study examining the perspectives of Navajo people on genetic research. Based on interviews with 45 individuals conducted from 2017 to 2018, a survey instrument was developed that asked 20 questions regarding knowledge, values, and beliefs about genetics and the moratorium, preferences for how biospecimens and data should be handled, and concerns about participating in genetics research. The survey was available in print and online to respondents and was administered between November 26, 2018 and March 18, 2019.

The survey included a brief description of the study and its purpose, definitions for various terms and a description of genetic research, a demographics questionnaire section, and questions about knowledge, values, experiences, the importance of various factors to be considered in a policy, and acceptability of potential research areas. Demographic questions included tribal affiliation, age, gender, religious or spiritual beliefs, highest level of educational attainment, and residential status (i.e., agency affiliation and residence in relation to the NN). In addition, the survey included multiple choice and Likert scale questions, adapted from previously collected interview questions, to explore attitudes and knowledge of the NN’s moratorium on genetic research, values and beliefs related to research (i.e., participation, biospecimen and data storage), concerns about genetic research, and recommendations for a genetic research policy and the moratorium. Lastly, open-ended questions allowed respondents to share additional thoughts related to genetics and the moratorium. The first open-ended question was “What comes to mind when you hear the word genetics?” and the second was “Do you have any further thoughts on the moratorium?” These questions were included to elicit any additional thoughts on genetics and the moratorium that were not covered in the survey, but respondents were not required to answer them. The survey was piloted with a trained survey developer, Navajo college students, and Navajo researchers. All initial survey respondents were invited to comment on any difficulties or concerns about the questions and these recommendations were included in the final survey. The survey is provided in Supplementary File S1.

Survey responses were manually entered into Microsoft Excel spreadsheets by two independent coders and entries were randomly verified by two other study team members. Categorical responses were converted to numerical values as a means of cleaning the data in Excel. Checking and correcting spelling errors, rearranging columns, and removing spaces were done manually by three members of the study team using Excel.

### Data Analysis

The demographic and survey data were first summarized by frequency and summary statistics (Supplementary Table S1). Frequency distributions were generated for questions related to concerns about genetic research involving Navajo people, the importance of aspects of a policy on genetic research, and types of genetic research that should be allowable on the NN. We examined the first open-ended question with a word cloud^2^ to describe the most common words elicited by the respondents. Free text responses to the second open-ended question (“further thoughts on moratorium”) were grouped into themes that were selected inductively based on an initial review of responses and refined through review by the broader study team. Themes were then applied to individual responses in an Excel spreadsheet and relevant quotes were extracted. This approach was reasonable given that responses were brief.

Association analyses were conducted in R Studio, v.1.3.959. As all of our survey data was composed of categorical variables, we used Pearson’s Chi-square test of association to evaluate the relationships between all relevant categorical variables in the survey. We partitioned data into contingency tables to examine trends and conducted Chi-square tests, with the alpha value corrected for multiple testing using a Bonferroni Adjustment (varied for each test). We tested the following categorical variables against 16 survey questions: residence on or off the NN, age, gender, highest level of completed education, religious or spiritual beliefs, agency affiliation, knowledge of the moratorium, and whether to lift the moratorium. For clarity, we separated respondent’s beliefs into the following four categories for data analysis: Navajo-based, Christian-based, mixed beliefs, and other. The Navajo-based beliefs responses included individuals who indicated traditional Navajo or Azeé Bee Nahaghá of the Diné Nation (ABNDN) views and beliefs. The Christian-based beliefs responses included individuals who indicated having Christian, Catholic, Mormon or other Christianity-based write-in options. The mixed beliefs responses included individuals who chose both a Navajo-based and Christian-based belief/view. The Other category included people who responded as Atheist, None of the Above, or any other belief that was written in that did not fit the previously described options. Unanswered questions (“NA”) were not considered and removed prior to analysis. Our null hypothesis was that the two variables were independent, and the alternative hypothesis was that the variables were dependent, or related in some way. A post hoc test, using residuals to test for cell significance, was conducted only after we found a statistically significant result (*p*-value adjusted for multiple testing) to determine where the differences came from and contribution to the *p*-value. If the post hoc analysis revealed an inconclusive relationship, this was noted in Supplementary Table S2. Inconclusive associations occurred in instances where the Chi-square value was largely being driven by the “no opinion” or “none/other” categorical variables.

## Results

A total of 742 surveys were completed (567 paper and 175 online). Surveys were excluded if respondents did not identify as Navajo (*n* = 15) or identified solely with another Indigenous tribe (*n* = 12), resulting in a total of 715 surveys from Navajo respondents. Of these, surveys were excluded if less than 75% of the questions (excluding demographic and open-ended questions) were not answered (*n* = 25). A total of 690 survey responses were included in subsequent analysis. Since we did not require an answer for the open-ended questions, 615 and 224 responses were analyzed for the first and second question, respectively.

The statistical analyses examined the relationship between respondent’s knowledge and willingness to participate in research in relation to age, gender, education, beliefs, agency affiliation, residence in relation to the NN, and the moratorium. A Chi-square analysis yielded several significant associations related to knowledge and participation in genetic research (Supplementary Table S2). Age, gender, beliefs, and agency affiliation resulted in few to no significant associations (*p*-value > Bonferroni corrected value). Residence, completed educational level, knowledge of the moratorium, and whether to lift the moratorium resulted in several significant associations which were further analyzed in a post hoc analysis. Post hoc analysis revealed inconclusive associations in 8 comparisons and these were excluded from further reporting. Specific results from the analysis are described in the sections below.

### Respondent Characteristics

**FIGURE 1:**
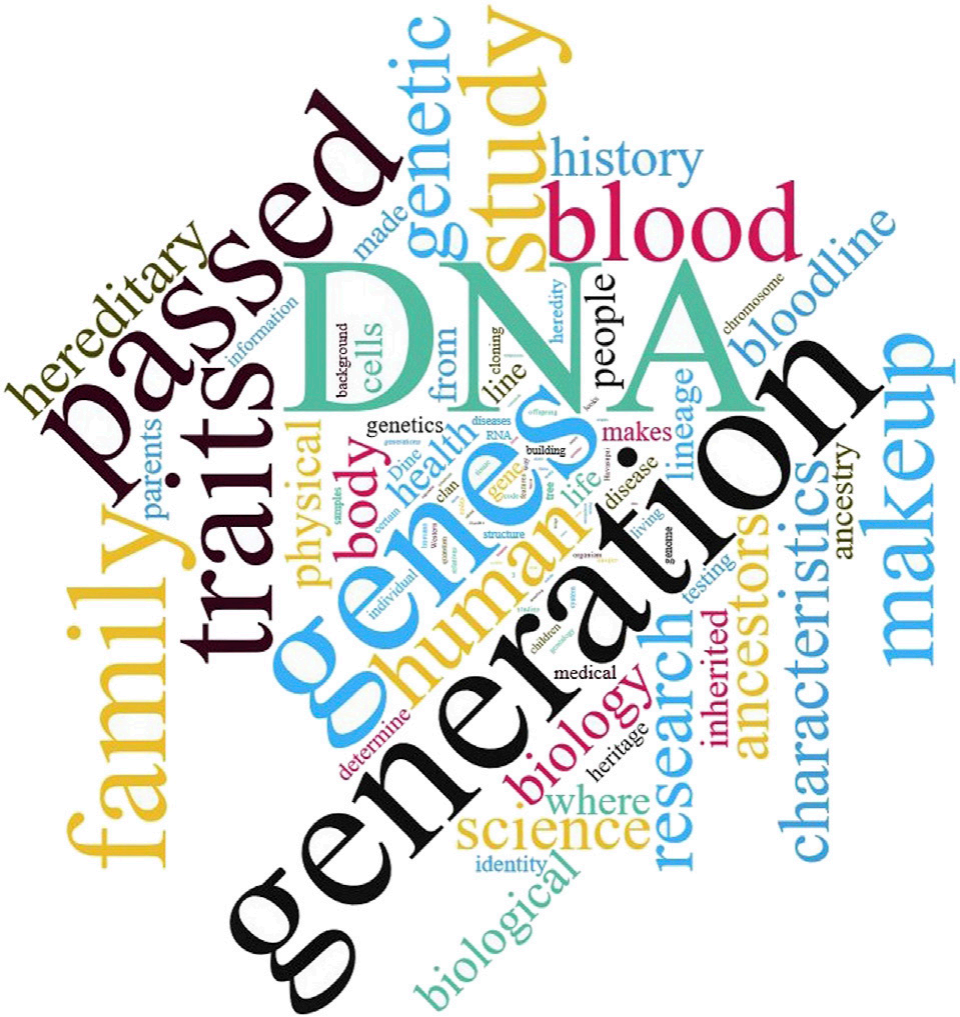
The most commonly-used terms to describe “genetics” by respondents. A word cloud was generated from respondent’s write-in responses to the question “What comes to mind when you hear the word genetics?” and the figure displays the words with highest frequency across all responses (*n* = 615). Common everyday words such as “a”, “the” and “and” were excluded from the wordlist automatically by the software and was manually double checked. The words with highest frequency are equivalent to the largest words displayed in the word cloud. Colors were randomly chosen for readability.

Table 1 shows summary statistics of the respondent demographics and characteristics. Most respondents were between the ages of 18 and 45 (58%), identified as women (64%), and reported having some college training (41%). When asked about spiritual or religious beliefs, 47% identified as having primarily Navajo-based beliefs, 24% as Christian-based, 14% reported a mix of beliefs, and 15% stated other or no beliefs. Survey respondents represented all 5 agencies on the NN and 76% reported that they primarily live on the NN. The majority of respondents did not know about the moratorium on genetic research before the survey (*n* = 422 or 63%). In general, the demographic characteristics of our survey participants included a higher percentage of women (64 vs 52%) and a higher number of respondents who lived primarily on the NN (76 vs 47%) than reported in the Navajo Population Profile from the 2010 Census data (Navajo Division of Health Navajo Epidemiology Center, 2013). The respondents most commonly thought of the following terms when asked about genetics: DNA, genes, generation, family, traits, blood, and passed down from ancestors/parents/grandparents/relatives (Figure 1).

**TABLE 1:** Characteristics of Navajo respondents to the survey.

Sociodemographic characteristics^a^	All respondents N (%)
**Age: (*N* = 690)**	
18–30	211 (31)
31–45	189 (27)
46–60	176 (26)
61–75	100 (14)
76+	14 (2)
**Gender: (*N* = 689)**	
Man	218 (32)
Woman	443 (64)
Two Spirit/LGBTQ/Other	28 (4)
**Education: (*N* = 687)**	
Middle schoo l - high school diploma/GED	142 (21)
Some college	282 (41)
Bachelor’s or vocational degree	135 (20)
Master’s, doctorate, or other professional degree	128 (19)
**Beliefs^b^: (*N* = 686)**	
Navajo-based	319 (47)
Christian-based	167 (24)
Mixed	96 (14)
Other	104 (15)
**Navajo Nation Agency Affiliation: (*N* = 678)**	
Northern	172 (25)
Western	135 (20)
Eastern	121 (18)
Central	113 (17)
Fort Defiance	68 (10)
Not sure	69 (10)
**Residence in relation to Navajo Nation: (*N* = 690)**	
On the Navajo Nation	521 (76)
Off of the Navajo Nation	102 (15)
Both (Transitory)	61 (9)
None	6 (1)
**Knowledge of the moratorium (before survey): (*N* = 670)**	
Yes	169 (25)
No	422 (63)
Don’t know	79 (12)

a Individuals who selected “prefer not to answer” were excluded.

b For clarity, we separated respondent’s beliefs/views into the following four categories for data analysis: Navajo-based, Christian-based, mixed beliefs, and other. The Navajo-based beliefs/views responses included individuals who had primarily traditional Navajo or Azeé Bee Nahaghá of the Diné Nation (ABNDN) views and beliefs. The Christian-based beliefs responses included individuals who had primarily Christian, Catholic, Mormon or other Christianity-based write-in options. The mixed beliefs responses were individuals who chose both a Navajo-based and Christian-based belief/view. The Other category included people who responded as Atheist, None of the Above, or any other belief that was written in that did not fit the previously described options.

### Concerns About Genetic Research

When asked about concerns related to genetic research involving Navajo tribal members, there was a consistent trend with the majority of respondents (∼81%) indicating that trusting researchers, transparency of the research process, research being done in an ethical way, privacy and confidentiality, benefits to their family, community or tribe, inclusion of their cultural beliefs, equitable access to genomic resources, and health and social justice were important to them (Table 2). A smaller percentage of respondents indicated that these were not important concerns (∼8%) or had no opinion (∼10%). Across the free text responses (224 comments), the most common broad categories of factors were (in order of frequency): 1) Needing more information on genetics and the moratorium, 2) conflicted feelings about the moratorium and genetic research, 3) importance of health research, 4) incorporation of Navajo cultural teachings, and 5) concerns about research ethics (Table 3).

**TABLE 2:** Respondents concerns about genetic research involving Navajo people.

Key concerns	Response		
	Not important^a^ N (%)	Important^b^ N (%)	No opinion N (%)
Trusting researchers (*N* = 689)	71 (10)	555 (81)	63 (9)
Transparency of research process (*N* = 689)	61 (9)	557 (81)	71 (10)
Research being done in an ethical way (*N* = 685)	60 (9)	558 (81)	67 (10)
Privacy and confidentiality (*N* = 684)	42 (6)	586 (86)	56 (8)
Benefits to my family, community or tribe (*N* = 688)	46 (7)	574 (83)	68 (10)
Inclusion of my cultural beliefs (*N* = 685)	64 (9)	542 (79)	79 (12)
Equitable access to genomic resources (*N* = 688)	63 (9)	534 (78)	91 (13)
Health and social justice (*N* = 688)	50 (7)	558 (81)	80 (12)

a Responses: Not at all important, Slightly important.

b Responses: Important, fairly important, very important.

**TABLE 3:** Themes and respondent’s quotes about the moratorium and genetic research.

Broad categories from overall responses	Respondent’s quotes
Need more information and education on genetics and the moratorium	*“We need to have more information on this subject*.*”*
	*“Eventually I can see the moratorium being lifted*, *but there are still a lot of unethical researchers and [the] community [is] still not knowledgeable about research issues to make the right decisions*. *Need more education in the community about issues related to research*.*”*
	*“I never know [sic] there was a moratorium*. *Perhaps educating the community would be beneficial at community level*.*”*
	*“I wonder why it’s been this long and even I wasn’t aware of it*.*”*
Conflicted about the moratorium and genetic research	*“Moratorium [on] genetic research [was] implemented for a reason*. *Lifting may cause disparities of misuse*.*”*
	*“I think lifting the ban will benefit the Navajo people*.*”*
	*“This isn’t a simple question of ‘yes*, *it should be lifted’ or ‘no*, *it should not be lifted*.*’ The NN need to have the proper staff*, *resources*, *policies*, *procedures*, *and infrastructure in place to exercise appropriate oversight and to protect our people*. *Without those in place*, *I would not support a lift of the moratorium*. *I support genetic research but only if it’s done in an ethical manner*, *with proper consent and transparency with the tribe/people/participants involved*, *and for their benefit*.*”*
	*“I am torn between the benefits that genetic research would provide with the potential for that information being used to discriminate against individuals for things like health insurance*. *Information has to be protected and not utilized as a justification for discriminatory practices*.*”*
Importance of health research to Navajo people	*“I think research done in the Navajo Nation could be very beneficial in understanding the disease patterns that so many of our people fall into*, *however*, *only if it uses the community-based participant research approach*, *meaning it connects with the tribe and asks what they want from the researching [sic] and keeping them involved every step of the way*.*”*
	*“I would and have participated in health research that did not require specimens*. *I feel that giving away a piece of me*, *however helpful it may be to ‘research’ and to bettering our Native community*, *there will always be that one person or researcher who will use it for harm or not understand the respect these samples should be treated with*.*”*
	*“I think genetic research related to health is important for a population as small as ours*. *I don’t think genetic research for molecular anthropological and similar research is important*.*”*
	*“Medical issues that may be caused by uranium exposure is of particular importance to me*. *I have a lot of autoimmune diseases and always wonder if its hereditary or caused by uranium exposure*.*”*
	*“If studies help end diabetes*, *alcoholism*, *obesity*, *it helps*.*”*
	*“If this research can help reduce the risk of cancer*, *I would fully recommend [this] research to be done*.*”*
Incorporation of and respect for Navajo cultural teachings	*“With strong policy and regulations in place*, *the moratorium should be lifted so that research can be done to address the monsters ailing our people*. *Diné people have the benefit of using both traditional and western medicine to address illness*. *It shouldn’t be exclusive*, *one way or the other*. *Wise people would say*, *meet your prayers halfway*.*”*
	*“There is no reason for this research*. *We have our medicine and our culture*.*”*
	*“The moratorium should be lifted but if genetic research was to happen on the Navajo Reservation or to any other Native group*, *the respect for cultural boundary*, *knowledge and respect should be practiced in a positive ethical way*.*”*
	*“There should be considerable consultation with medicine healers and the decision to lift or not*, *they should have major say*.*”*
Concerns about research ethics	*“Navajo Nation should be transparent to allow how diseases could be controlled through a study that can benefit the health of all especially in the 21st century*.*”*
	*“…data misuse*, *data breaches*, *breaches in confidentiality*, *breaches of third party misuse of data*, *exposure of the participants genetic information*, *and risk of exposure to allow other researchers (private/governmental) agencies to collect and reproduce or replicate genes to fit another propaganda is likely*.*”*
	*“Although I am optimistic about genetic research and it’s benefits*, *I am concerned about privacy and protecting our Navajo people*.*”*
	*“Controlling ownership of results of research by Navajo Nation*.*”*

The categories are listed in order of decreasing number of responses (top to bottom).

### Knowledge of the Moratorium and Research

The survey asked 7 questions, in which some responses were captured on a Likert scale, that related to respondent’s knowledge of various topics including the following: knowledge of the moratorium, research process or scientific method, genetic science, discussions by the NN about genetic research, the lawsuit filed by the Havasupai Tribe against Arizona Board of Regents about a research study conducted at Arizona State University (ASU) (hereinafter referred to as the “lawsuit filed by the Havasupai Tribe”), and positive and negative stories of research using biospecimens. Most respondents (63%) indicated that they had not heard about the moratorium on genetic research on the NN whereas 25% stated they knew about it and 12% responded “don’t know/unsure”. Of these, knowledge of the moratorium was statistically associated with where an individual resided in relation to the NN (*p*-value = 3.878e-05), age (*p*-value = 5.49e-05), educational level (*p*-value < 2.2e-16), and opinions related to lifting the moratorium (*p*-value = 1.408e-08). In the post hoc analysis, individuals who primarily resided off the NN reported higher rates of knowing about the moratorium as well as hearing about discussions of genetic research by the NN, the lawsuit filed by the Havasupai Tribe, and negative stories about biospecimens used in research. In comparison, those who lived on the NN or were transitory did not have statistically significant differences from those groups who knew about the moratorium, genetic research discussions, the lawsuit filed by the Havasupai Tribe, and negative stories.

A Chi-square test of independence revealed that among individuals who were familiar with the research process or scientific method, there was significant association with age range (*p*-value = 2.85e-05), educational level (*p*-value = 1.31e-14), knowledge of the moratorium (*p*-value < 2.2e-16), and opinions related to lifting the moratorium (*p*-value = 2.79e-05). Post hoc comparisons revealed that individuals with a masters or doctorate degree had higher rates of knowing about the research process. In comparison, knowledge of the research process was statistically similar among respondents with different educational levels. There was also a positive association between an individual having a high school degree or equivalent and having a higher rate of answering “don’t know” about the moratorium. Being knowledgeable about genetic science was statistically associated with knowledge of the moratorium (*p*-value = 4.871e-05).

Individuals who were knowledgeable about discussions of genetic research on the NN, the lawsuit filed by the Havasupai Tribe, and hearing about positive and negative stories of research using biospecimens were all strongly associated with where an individual resided in relation to the NN (*p*-value = 0.0003564, 4.231e-14, 0.00278, and 1.167e-13, respectively), educational level (*p*-value = 1.496e-07, <2.2e-16, 9.767e-07, and 3.558e-16, respectively), and previous knowledge of the moratorium (*p*-value < 2.2e-16, <2.2e-16, 8.643e-13, and <2.2e-16, respectively). There was also a strong association between individuals who were knowledgeable about discussions of genetic research on the NN as well as hearing about positive (*p*-value = 0.0006243) and negative stories (*p*-value = 1.57e-09) of research using biospecimens and opinions related to lifting the moratorium (*p*-value = 4.091e-06). Post hoc comparisons revealed that individuals with a masters or doctorate degree had higher rates of hearing about discussions of genetics on the NN, lawsuit filed by the Havasupai Tribe, and positive and negative stories of medical research using biospecimens. In comparison, other educational levels did not show statistically significant differences.

### Participation in Genetic Research

The survey asked 7 questions related to attitudes about participating or donating biospecimens in a research study and responses were captured on a Likert scale: participation and donation of biospecimens in relation to beliefs, data sharing and donation of biospecimens in relation to knowing the researcher’s background, community involvement, approval by community leaders, and if the disease under study affected the community. Of these, when asked if their beliefs would impede them from participating in genetic research or donating biospecimens, there were no statistical associations with where a respondent resided or their age, gender, educational level, agency affiliation, and knowledge of the moratorium. There were significant associations with attitudes about donation of biospecimens with religious beliefs (*p*-value = 1.102e-12), and whether to lift the moratorium with attitudes about donation of biospecimens (*p*-value = 6.619e-15) and with participating in genetic research (*p*-value < 2.2e-16). Post hoc comparisons revealed that individuals who stated they had Navajo-based beliefs also had higher rates of strongly agreeing that their beliefs would prohibit them from donating biospecimens for research. In a post hoc analysis, individuals who did not support lifting the moratorium also had higher rates of strongly agreeing with not participating in research or donating biospecimens because of their beliefs.

When asked about their willingness to participate in research under specific criteria (i.e., community involvement, community leader review, studying a disease affecting the community), a Chi-square test of independence revealed that there were statistically significant associations with where people resided in relation to the NN (*p*-values < 0.001), educational attainment (*p*-values < 0.001), knowledge of the moratorium (*p*-values < 0.001), and opinions on whether to lift the moratorium (*p*-values < 0.001). In contrast, there were no differences associated with age, gender, beliefs, or agency affiliation. Post hoc analyses revealed that respondents residing off the NN had a higher rate of responding that they were very likely to donate biospecimens if community members were involved in the research process, if the research was approved by community leaders, and if a disease that affected their community was being studied. In addition, a post hoc analysis revealed that individuals who indicated “don’t know” about the moratorium also had a higher rate of having no opinion about community involvement, community leader review, studying a disease affecting the community, and sharing data.

**FIGURE 2:**
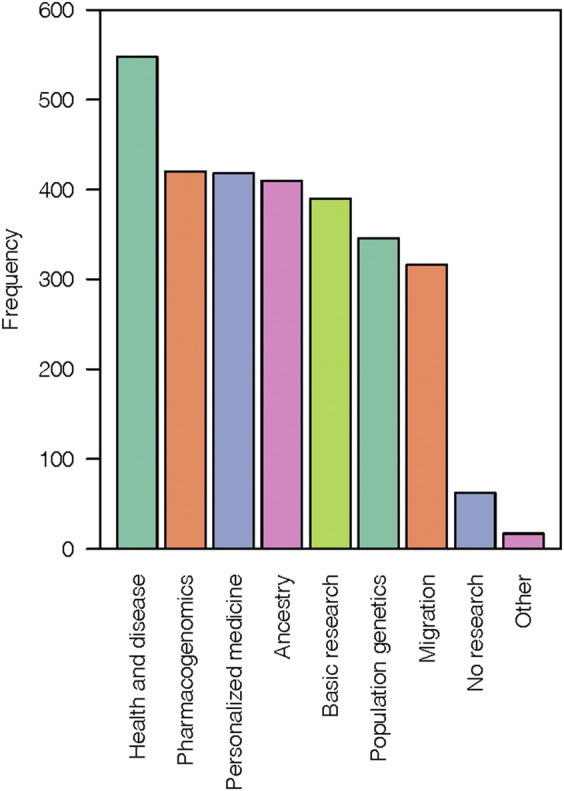
Types of genetic research that should be allowable on the Navajo Nation. The bar plot shows the frequency of respondent’s answers for each provided research category. Respondents were allowed to choose multiple answers for this question. The “Other” category included write-in responses like human microbiome, nutrigenetics, longevity, clan characteristics, and cancer.

When asked what types of genetic research should be allowable on the NN, respondents were able to choose multiple research topics based on a general category title and descriptive text. Respondents who chose all research category options (*n* = 216) were interpreted to be favorable of allowing those types of genetic research on the NN. When examined further, research related to health and disease (*n* = 548), pharmacogenomics (*n* = 420), personalized medicine (*n* = 418) and ancestry (*n* = 410) were highest in frequency compared to other research topics (Figure 2, Supplementary Table S3). Lowest in frequency were population genetics (*n* = 346) and migration research (*n* = 316). Finally, 62 respondents did not choose any of the options presented.

### Opinions Regarding the Moratorium and Research Policy

People’s views and opinions on lifting the moratorium and developing a future research policy are of great importance to Navajo policy makers so we asked whether respondents thought that the moratorium of genetic research should be lifted, thus allowing some genetic research to involve Navajo people on the NN. The majority of respondents (46%, *n* = 316) were “not sure” if the moratorium should be lifted while 36% (*n* = 251) of respondents said that the moratorium should be lifted and 18% (*n* = 122) said that the moratorium should not be lifted. There were no statistically significant results related to where respondents resided, age, gender, educational level, beliefs or agency affiliation on whether or not to lift the moratorium.

Responses to lifting the moratorium were categorized by “yes”, “no”, and “not sure” and Chi-square tests of independence revealed that opinions on the moratorium were statistically associated with whether an individual would participate in research if they knew a researcher’s background, community involvement, community leader approval, diseases affecting the community, and data sharing (*p*-values were all <2.2e-16). Of these, post hoc associations revealed that there were two primary groups that showed associations with opinions on whether to lift the moratorium on the NN. First, individuals who supported lifting the moratorium were also very likely to donate biospecimens if they knew a researcher’s background and if the project had community involvement, community leader approval, focused on diseases affecting the community, and data sharing. Second, in contrast, individuals who did not support lifting the moratorium were very unlikely to donate biospecimens even if they knew a researcher’s background, or if the project had community involvement, community leader approval, focused on diseases affecting the community, and data sharing.

If the NN decides to lift the moratorium, then a genetic research policy will need to be adopted by the tribe. When respondents were asked about what a genetic research policy on the NN should include, over 80% of respondents reported that tribal oversight, incorporation of cultural knowledge, benefits to the Navajo tribe, community engagement, and data sharing protections were important factors (including responses of important, fairly important, and very important) in a future research policy (Table 4). Finally, 71% of respondents indicated that it was important that researchers conducting the research should be Navajo.

**TABLE 4:** Importance of factors for a genetic research policy.

Key concepts	Responses N (%)					
	No opinion	Not at all	Slightly important	Important	Fairly important	Very important
Tribal oversight of research (*N* = 683)	68 (10)	31 (5)	41 (6)	136 (20)	107 (16)	300 (44)
Inclusion of cultural knowledge (*N* = 683)	58 (8)	14 (2)	22 (3)	135 (20)	74 (11)	380 (56)
Research benefits to Navajo tribe (*N* = 685)	62 (9)	13 (2)	18 (3)	128 (19)	78 (11)	386 (56)
Researchers should be Navajo (*N* = 684)	90 (13)	40 (6)	69 (10)	135 (20)	99 (14)	251 (37)
Community engagement a part of the research (*N* = 682)	65 (10)	20 (3)	33 (5)	150 (22)	82 (12)	332 (49)
Data sharing protections in place (*N* = 682)	69 (10)	13 (2)	20 (3)	117 (17)	59 (9)	404 (59)

## Discussion

To our knowledge, this is the first survey of perspectives attitudes, concerns, and knowledge about genetic research in the Navajo community. This survey offers a snapshot of the Navajo community’s views about genetic research and does not represent the entire tribes perspectives, as this is a complex issue. As genetic research continues to be considered and debated, it will be important to develop guidelines and policies to govern how research is conducted in ways that are acceptable to all Navajo people. Many Indigenous peoples, including Navajo, have expressed hesitation to participate in research against a backdrop of historical missteps and concerns about “helicopter science,” such as the Human Genome Diversity Project’s failed attempts to survey Indigenous peoples worldwide (TallBear, 2007) or the Havasupai Tribe learning that DNA samples that were intended for diabetes research were instead used to study schizophrenia and human migration (Drabiak-Syed, 2010; Mello and Wolf, 2010; Garrison, 2012). Although the Navajo Nation enacted the moratorium on genetic research before the Havasupai Tribe learned of the misuse of DNA samples, it is conceivable that discussions about genetics were delayed until there was resolution about the lawsuit that the Havasupai Tribe filed. On the other hand, there is great promise with the emergence of pharmacogenomics research with tribal partners and increasing attention on precision health and precision medicine initiatives that may offer benefits to all people, including Indigenous peoples. Therefore, it is essential to understand the perspectives and concerns of Navajo community members at a time that a genetics policy is being discussed and developed and general discussions are taking place about the future potential benefit of genetic research to Navajo people.

While respondents differed in their perspectives about several important issues, our findings show that the majority of people did not know about the moratorium prior to being invited to complete the survey and the majority of respondents were not sure if the moratorium should be lifted. Interestingly, age, gender, educational attainment, religious or spiritual beliefs, and location (agency affiliation and residence relative to NN) did not impact people’s opinions on whether the moratorium should be lifted or not. This is not entirely surprising given the moratorium was put in place in 2002 (Gift of Life, 2002) and relatively few large public discussions have taken place since. This suggests that more public education about genetic research, its potential risks and benefits, and plans for governance and oversight might offer more opportunities for community members to become informed and formulate an opinion. This was apparent from the responses provided in the second open-ended question.

Of those who knew about the moratorium, respondents were more interested in participating in genetic research studies and would donate their biospecimens to a study under certain conditions. Our results showed that those who knew about the moratorium tended to have higher educational attainment, were more informed about genetics, the research process, and ongoing discussions about genetics on Navajo and the lawsuit filed by the Havasupai tribe. Those who knew about the moratorium were also more likely to support lifting the moratorium. Since 2017, there have been public workshops, discussions, and radio forums, some of which were venues in which we recruited participants, that have likely contributed to increased knowledge and awareness of genetics and its associated concerns (NIH Tribal Health Research Office, 2017). The moratorium was mentioned in each of the public talks; however, the moratorium itself was not the focal point in these public engagements. In the open-ended comment section of the survey, several respondents mentioned having taken college-level courses in genetics, seeing advertisements and opportunities to participate in direct-to-consumer genetic testing or research studies, and wanting to participate in genetic studies that could offer benefits to their families.

Overall, relatively few respondents felt knowledgeable about genetics, though we expected higher frequency given 1) Navajo people monitor familial relationships through k’e, a complex genealogical clan system that delineates how extended family members are related to each other (Nielson and Zion, 2005), 2) there is a history of genetic research being conducted with Navajo people prior to the moratorium (Begay et al., 2020), and 3) there are concerns about the effects of radiation exposure from uranium mining that may contribute to high cancers rates across the NN (Brugge and Goble, 2002). However, in each of these cases, discussions about genetics with the larger community have not necessarily been a focal point.

The concerns mentioned by Navajo respondents were similar to those voiced by other American Indian (Chadwick et al., 2019) and Alaska Native (Hiratsuka et al., 2020a; Hiratsuka et al., 2020b) communities, namely that considerations for effective and socially responsible research partnerships should be taken into consideration. Past unethical practices which affected tribal communities (Bowekaty and Davis, 2003; Mello and Wolf, 2010) continue to drive many of the concerns that the Navajo community voiced. For example, in the open-ended comments (Table 3), Navajo respondents overwhelmingly wanted more education about genetic research and genetics-related health literacy and its potential implications for their communitie’s health and disease. Specifically, respondents described a need for more information about the moratorium, its history, and how a genetic research policy would be beneficial. Many were unaware of the moratorium and felt conflicted about answering until there was more education about the topic, which may explain the high frequency of the “not sure” responses.

For the Navajo respondents, there were expressed needs for further consultation at the individual, chapter, regional, and NN level as well as consultation with cultural leaders on these issues. The Diné people live by culturally oriented guidelines that were created to bring balance and harmony within the universe that surrounds the Diné people, broadly referred to as the Diné Fundamental Law (Austin, 2009). Navajo people seek guidance from spiritual leaders for many life issues and may hesitate or refuse to be involved in research that misaligns with their cultural values, including biomedical research with biospecimen donation. The NN has two non-profit organizations known as the Diné Medicine Men Association, Incorporation (Diné Bi Nahagha’ Yee Da’ahótą’) and the Diné Hataałii Association, Incorporation (Diné Be’azee’ííłʼíní Yee da’ hótą́) that comment on matters regarding Navajo customs and speak with authority and authenticity on Navajo traditional healing and customs. Cultural leaders and organizations like this should be involved in consultation when and if the moratorium is lifted should be involved in the creation of a genetic research policy, especially as they were deeply involved in previous discussions regarding the placement of the moratorium (Gift of Life, 2002).

At the national level, there is an increase in community and tribally-based participatory research approaches to genetics and genetics-related research (Woodbury et al., 2019; Hiratsuka et al., 2020c), ethical research frameworks (Claw et al., 2018), and tribal control over health-related research (Hiratsuka et al., 2017; Around Him et al., 2019; Chadwick et al., 2019). These are attempts to rectify the concerns expressed by American Indian communities. In particular, for genomic data, the unrestricted access to genomic data (Hudson et al., 2020) and recruitment of tribal members from urban areas who are living off-tribal lands (James et al., 2018; Tsosie et al., 2019) pose critical issues for tribes.

### Limitations and Future Directions

The goal of our study was to assess the Navajo public’s perspectives about genetic research. The survey specifically gathered information on familiarity with the moratorium on genetic research studies, identified types of genetic research that the community would allow, and sought to understand their willingness to donate biospecimens for research under different scenarios. The order of questions and information given in the survey could have influenced how a respondent answered questions. For example, 75% of respondents indicated not knowing about or being unsure if they knew about the moratorium, then they were asked to indicate the types of genetic research they would support and weigh out factors that would influence their decisions to participate in genetic research. At the end of the survey, nearly 36% of respondents indicated they would support lifting the moratorium on genetic research and 46% indicated they were not sure. The survey asked about participation in research if it was focused on diseases of relevance, various factors that influenced participation, and considerations for a genetic research policy, thus, respondents may have been influenced by the questions that were posed and the finite multiple-choice options. In addition, a definition was given for genetic research and later were asked to indicate what words comes to mind when they hear the word genetics. In this case, the results suggest that the survey had prompted several common words that they might not have thought of on their own.

Another limitation of our study is that the surveys were conducted using a convenience sample where people were approached in person at various events and through online social media and email. Thus, this is not a representative sample of the entire community. There was an overrepresentation of survey responses from people within the Northern Agency where many surveys were completed in person. On the other hand, online surveys may have been completed primarily by people who have access to social media (where the survey was advertised), the internet, or a computer device and are mostly living in urban areas or off the reservation. Respondents may have different views and varying access from those living on the reservation. Many elders do not have access to the internet or social media; therefore, we may have potentially left out their perspectives. While the house-to-house approach and recruiting at agency council meetings and other public events allowed us to reach a wider range of the Navajo public than just an online survey, knowledge of genetics may not have been adequately captured due to how the questions were worded and because Navajo people have a strong history of oral storytelling (Shreve, 2014) such that many people may prefer to discuss and elaborate on genetic concepts verbally instead of with a survey. Another limitation is that the survey was written and administered in English, potentially leaving out elders, cultural leaders, and people who spoke primarily Navajo. The survey does not allow us to determine which concerns or beliefs are viewed as most important or how much spiritual and religious beliefs influence public views on genetics. Finally, the survey offered Christian, Catholic, and Mormon as three of multiple religious beliefs options, but did not explicitly define “Christian” in the survey. We collapsed the categories under a broader “Christian” category for the purposes of our analysis and did not do further analyses within this category.

The future directions would include involving a range of community members in in-depth discussions about genetic research. Many respondents felt that cultural leader input was needed, and future discussions could focus on Navajo cultural leader perspectives in a culturally appropriate setting. In addition, a more refined survey could be replicated in multiple types of Navajo stakeholder populations and increasing the number of individuals in the survey as well as translating the survey into the Navajo language. For elders or those who have trouble understanding written Navajo, an oral survey option could be provided.

## Conclusion

This study is the first to empirically assess perspectives and attitudes towards genetic research in the general Navajo population. The respondents in our study felt that more information and education was needed on this topic before making decisions about lifting the moratorium or participating in genetic research. Additionally, they felt that cultural leaders and various levels of the community (chapter and agency) should be consulted. While there was interest in the potential of genetic research to study the underlying causes of disease and morbidities in the community, there were also concerns related to tribal oversight, data sharing, sample storage and acquisition and cultural considerations. This study should be viewed as a snapshot of the Navajo population’s perspectives and can serve and as a baseline for future studies that assess a tribal community’s views and opinions regarding genetic research. As the NN continues to reconsider the moratorium on genetic research, additional studies may be helpful to validate the observations in this study.

## Data Availability

The datasets presented in this article are not readily available because the raw data supporting the conclusions of this article is the under the purview of the Navajo Nation Human Research Review Board and is subject to their approval. Requests to access the datasets should be directed to katrina.claw@cuanschutz.edu.
