# Supplementary material for: Perspectives on Genetic Research: Results From a Survey of Navajo Community Members
Source: Front Genet. 2021 Dec 2;12:734529. doi: 10.3389/fgene.2021.734529 (PMC8675633; doi:10.3389/fgene.2021.734529)
Supplement: Supplementary file 1 [file DataSheet1.PDF]

UNIVERSITY of WASHINGTON

Seattle Children's HOSPITAL • RESEARCH • FOUNDATION | Treuman Katz Center for Pediatric Bioethics

DINÉ POLICY INSTITUTE

Perspectives on Genetics in the Navajo Nation Survey

PURPOSE: We are conducting a survey about Navajo opinions and perspectives on genetic research. The overall results from this survey will be used to inform the development of a policy relating to genetic research on the Navajo Nation. Your participation in this interview is completely voluntary. Any information revealed on the survey will be kept anonymous. The survey will take approximately 5-10 minutes to complete, and you are welcome to enter into a raffle drawing at the end of the survey. At the end of the survey study, we will raffle off twenty gift cards in the amount of $25 for survey participants. Thank you for your time. Ahéhee'!

DEMOGRAPHICS:

What tribe(s) are you enrolled with? Please check all that apply.

- ☐ Diné (Navajo) Nation
- ☐ Other tribe(s), please list: _______________________
- ☐ Other ethnicity, please list: _________________
- ☐ I am not Native American.

Do you currently live on the reservation?

- ☐ I primarily live on the Navajo Nation
- ☐ I primarily live off of the Navajo Nation (e.g., Urban)
- ☐ I am transitory, I live on and off the Navajo Nation
- ☐ None of the above

Age:

- ☐ 18-30
- ☐ 31-45
- ☐ 46-60
- ☐ 61-75
- ☐ 76+

How do you identify?

- ☐ Man
- ☐ Woman
- ☐ Two Spirit/ LGBTQ (Lesbian, Gay, Bisexual, Transgender, or Queer)
- ☐ Other:_____________

What is the highest level of education you have completed?

- ☐ Middle school
- ☐ Some High School
- ☐ High School Diploma/ GED
- ☐ Vocational School
- ☐ Some College
- ☐ Bachelor's Degree
- ☐ Master's Degree
- ☐ Professional Degree (JD, RN, MD)
- ☐ Doctorate Degree

What is your current religion, if any? Please check all that apply.

- ☐ Traditional Navajo
- ☐ Azee' Bee Nahagha (Native American Church)
- ☐ Christian
- ☐ Catholic
- ☐ Mormon
- ☐ Atheist
- ☐ None of the above
- ☐ Other: ________________

Which agency are you affiliated with?

- ☐ Western
- ☐ Central
- ☐ Northern
- ☐ Eastern
- ☐ Fort Defiance
- ☐ Not sure/Not applicable

Please provide your Chapter affiliation: _______________________________________

UNIVERSITY of WASHINGTON

Seattle Children's HOSPITAL • RESEARCH • FOUNDATION | Treuman Katz Center for Pediatric Bioethics

DINÉ POLICY INSTITUTE

Perspectives on Genetics in the Navajo Nation Survey

DEFINITIONS AND BACKGROUND:

Genetic research: The study of genes (DNA instructions) and how traits are passed from one generation to the next.

Biological specimen or biospecimen: Samples of material, such as urine, blood, tissue, cells, DNA, RNA, and protein from humans, animals, or plants.

Moratorium: A temporary ban or a stopping of an activity for an agreed amount of time.

Background the Navajo Nation's moratorium on genetic research: After consultation with the Navajo Nation's Health and Human Services committee, tribal leaders, traditional healers, and Navajos with scientific training, the Navajo Nation recognized a lack of policies or guidelines for genetic research protocols. In April 2002, the Health and Human Services committee of the Navajo Nation approved a "moratorium of genetic research studies conducted within the jurisdiction of the Navajo Nation until such time that a Navajo Nation Human Research Code has been amended by the Navajo Nation Council".

1. What comes to mind when you hear the word, "genetics"?

2. Before today, did you know about the Navajo Nation's moratorium on genetic research?

☐ Yes ☐ No ☐ Don't know

KNOWLEDGE, VALUES, AND BELIEFS	Strongly Disagree	Somewhat Disagree	Neutral/No Opinion	Somewhat Agree	Strongly Agree
3. I am familiar with the research process and/or the scientific method.	☐	☐	☐	☐	☐
4. I am more knowledgeable about genetics than other people.	☐	☐	☐	☐	☐
5. I have strong spiritual or religious beliefs.	☐	☐	☐	☐	☐
6. Because of my spiritual values, I would not want to participate in genetic research.	☐	☐	☐	☐	☐
7. My spiritual values would prohibit me from donating biospecimens for research.	☐	☐	☐	☐	☐

KNOWLEDGE OF RESEARCH AND MORATORIUM	Yes	A little bit	No	No Opinion
8. Have you heard of any discussions about genetic research by the Navajo Nation government?	☐	☐	☐	☐
9. Have you heard about the Havasupai Tribe's case with Arizona State University relating to genetics?	☐	☐	☐	☐
10. Have you heard *positive* stories about medical research using biospecimens (e.g., samples of saliva, DNA, or tissue)?	☐	☐	☐	☐
11. Have you heard *negative* stories about medical research using biospecimens. (e.g., samples of saliva, DNA, or tissue)?	☐	☐	☐	☐

Perspectives on Genetics in the Navajo Nation Survey

BIOSPECIMENS AND DATA	Very Unlikely	Somewhat Unlikely	Possibly	Somewhat Likely	Very Likely	No Opinion
12. How likely are you to donate your biospecimen if you knew the researcher's background and training?	☐	☐	☐	☐	☐	☐
13. How likely are you to donate your biospecimen if your community members helped develop the genetic research study?	☐	☐	☐	☐	☐	☐
14. How likely are you to donate your biospecimen if the study was reviewed and approved by your community leaders?	☐	☐	☐	☐	☐	☐
15. How likely are you to donate your biospecimen if what was being studied was a disease that affected your community? (e.g. cancer, diabetes)	☐	☐	☐	☐	☐	☐
16. How likely are you to participate in a study if your data is shared with other researchers?	☐	☐	☐	☐	☐	☐

17. How important to you are the following concerns about genetic research involving Navajo people?

	Not at all Important	Slightly Important	Important	Fairly Important	Very Important	No Opinion
Trusting researchers	☐	☐	☐	☐	☐	☐
Transparency of research process	☐	☐	☐	☐	☐	☐
Research being done in an ethical way	☐	☐	☐	☐	☐	☐
Privacy and confidentiality (e.g., identity, data)	☐	☐	☐	☐	☐	☐
Benefits to my family, community or tribe	☐	☐	☐	☐	☐	☐
Inclusion of my cultural beliefs	☐	☐	☐	☐	☐	☐
Equitable access to genomic resources (e.g., diagnostic tests)	☐	☐	☐	☐	☐	☐
Health and social justice	☐	☐	☐	☐	☐	☐
Other: ________________	☐	☐	☐	☐	☐	☐

Continued on the next page

Perspectives on Genetics in the Navajo Nation Survey

18. How important is it to include the following parts in a policy on genetic research?

	Not at all Important	Slightly Important	Important	Fairly Important	Very Important	No Opinion
Tribal oversight	☐	☐	☐	☐	☐	☐
Cultural knowledge	☐	☐	☐	☐	☐	☐
Benefit to Navajo tribe	☐	☐	☐	☐	☐	☐
Researchers should be Navajo	☐	☐	☐	☐	☐	☐
Community engagement	☐	☐	☐	☐	☐	☐
Data sharing protections	☐	☐	☐	☐	☐	☐
Other: ________________	☐	☐	☐	☐	☐	☐

19. What types of genetic research should be allowable on the Navajo Nation? (*Please check all that apply.)*

- ☐ Studying one's family or ethnic descent (*ancestry research*)
- ☐ Studying how people have moved around the world (*migration research*)
- ☐ Studying how genes affect a person's response to drugs (*pharmacogenomics*)
- ☐ Studying genes related to health and disease (*e.g., diabetes, cancer, heart disease, etc.)*
- ☐ Studying medical care customizable for individual patients (*precision/personalized medicine*)
- ☐ Studying genetic differences within and between populations (*population genetics*)
- ☐ Studying the basics of genetics to gain greater knowledge (*basic research*)
- ☐ Other: ___________________

20. Should the Navajo Nation lift the moratorium (ban) on genetic research?

☐ Yes ☐ No ☐ Not sure

21. Do you have any further thoughts on the moratorium?

22. Would you like to enter into the raffle drawing? If yes, please fill out the contact information below. Your contact information will be separated from the survey.

☐ Yes ☐ No

Ahéhee' (Thank you) for your time!

Yes, please enter me into the raffle drawing!

Name: _________________________________
Address: _______________________________
____
Email: _________________________________
Phone number: __________________________

At the completion of the survey study, 20 gift cards in the amount of $25 will be raffled off to interested survey participants! Ahéhee'!
